# Systematic Review of Methods for Measuring Circulating Cell-Free DNA in Plasma of Healthy Individuals

**DOI:** 10.3390/diagnostics16060821

**Published:** 2026-03-10

**Authors:** Aaron Das, Ilirjana Gocaj, Alisa Yurovsky

**Affiliations:** Department of Biomedical Informatics, Stony Brook University, Stony Brook, NY 11794, USA

**Keywords:** cell-free DNA (cfDNA), disease detection, early detection, biomarker, healthy controls

## Abstract

**Background/Objectives**: Standardizing measurement of circulating cell-free DNA (cfDNA) in healthy individuals is critical for its application as a reference in biomarker research, yet methodological variability remains poorly documented. **Methods**: We systematically reviewed 35 studies (*n* = 1250 healthy subjects) assessing how pre-analytical handling, extraction kits, and quantification methods influence plasma cfDNA levels. We identified quantification approaches (qPCR vs. fluorometry) and use of custom extraction kits as the strongest drivers of variability. **Results**: In qPCR studies, including ≥ 40 subjects reduced variability, underscoring the importance of adequate sample size. Commercial kits produced more consistent yields than in-house protocols; in our dataset, many studies used Qiagen’s QIAamp Circulating Nucleic Acid Kit, which has historically served as a widely used reference platform. Blood collection in EDTA tubes had minimal impact when commercial kits were used. **Conclusions**: Based on these findings, we recommend EDTA tubes, a standardized commercial extraction kit, and qPCR quantification to minimize cfDNA measurement variability in healthy cohorts. Finally, we provide expected cfDNA ranges for healthy individuals based on methodological flow, which can guide future benchmarking efforts and biomarker studies, improving comparability and early-detection research.

## 1. Introduction

Circulating cell-free DNA (cfDNA) consists of fragmented nucleic acids released by lysed cells into bodily fluids, such as blood (serum and plasma), urine, and saliva [1,2]. These nucleic acids have translocated beyond their region of origin and are observed in the sampled bodily fluid. cfDNA originates primarily from apoptotic and necrotic cell turnover, and its concentration in circulation is influenced by both physiological homeostasis and pathological conditions [3,4]. In healthy individuals, homeostasis typically maintains cfDNA at low levels but can still exhibit transient elevations in response to specific physiological stresses. For example, acute physical exercise has been shown to increase cfDNA concentrations significantly (up to 9.8-fold) immediately following strenuous activity before returning to baseline levels [5]. cfDNA release is often elevated when in a diseased state, especially cancer, due to increased cell death and impaired clearance mechanisms. This has led to increasing interest in cfDNA as a non-invasive biomarker for disease detection, prognosis, and monitoring through liquid biopsies.

While the diagnostic utility of cfDNA has been extensively studied in various diseases, especially cancers, there is a general lack of standardization regarding how cfDNA should be measured across studies [6,7,8]. Typically, measurements of cfDNA are influenced by numerous methodological variables, including pre-analytical factors (such as blood collection tube type and sample processing time), DNA extraction protocols, and quantification techniques [9,10]. However, even among healthy individuals, variation in these factors can lead to considerable inconsistencies of cfDNA yield. For example, cfDNA concentrations are generally higher in serum than in plasma due to leukocyte lysis during coagulation, which is why plasma is preferred for more specific cfDNA detection [9,10]. Similarly, variability in DNA isolation methods, such as the use of Qiagen vs. non-Qiagen extraction kits or custom in-house protocols, has been diligently reported in diseased individuals but remains poorly characterized in healthy populations [11,12,13]. An additional challenge in cfDNA standardization arises from quantification techniques. While fluorometric methods (e.g., Qubit, PicoGreen) measure total DNA content, quantitative PCR (qPCR) and digital PCR (dPCR) allow for more specific fragment analysis via use of specific primers [3]. The decision of which specific primers to use is another source of variance in measured cfDNA levels [3,14].

Despite the general lack of consensus, various systematic reviews have compiled the available information to make conclusions and recommendations for cfDNA measurements based on levels measured in individuals with pathological conditions. For example, Trigg et al. [10] found that qPCR and fluorimetry (PicoGreen^®^ or Qubit^®^) yielded comparable cfDNA levels in seven out of eight of the articles they assessed in terms of quantification, and Kumar et al. [11] recommends Qubit for its high sensitivity to low cfDNA concentrations.

Current systematic reviews focus on cfDNA measurements in diseased individuals, leaving methodological variability in healthy controls underexplored [11,12,13]. This is a critical limitation because establishing accurate and reproducible baseline cfDNA levels in healthy populations is essential for using cfDNA as a reliable comparator in biomarker research. Although total cfDNA alone has limited clinical utility as a standalone biomarker, establishing reproducible baseline measurements in healthy individuals is essential for studies of biologically and clinically relevant cfDNA subfractions, where total cfDNA measurement serves as an upstream, normalization, or quality-control component. Without clearly defined reference ranges and standardized methodologies in healthy controls, it is difficult to determine what constitutes a clinically meaningful elevation of cfDNA. This is especially important for early-detection strategies, where the ranges of healthy and diseased individuals may have a large overlap.

Thus, given methodological inconsistencies in cfDNA measurement, a systematic review is needed to characterize how cfDNA extraction, quantification, and pre-analytical handling influence reported cfDNA concentrations in healthy individuals. The objective of this review is to analyze existing studies measuring cfDNA levels in healthy individuals and to identify methodological factors associated with high variability in reported cfDNA concentrations. We investigate how the combination of specific extraction kits, quantification techniques, and pre-analytical conditions affect cfDNA variability in healthy controls, and we assess whether the existing literature supports movement toward greater methodological consistency.

## 2. Materials and Methods

The study protocol was registered with and approved by INPLASY^®^ (registration number: INPLASY202530125). We followed the Preferred Reporting Items for Systematic reviews and Meta-Analysis (PRISMA) statement [15].

### 2.1. Literature Search and Selection Criteria

A publication search was carried out in Google Scholar, PubMed, Scopus, and Embase between July 1, 2024 and August 6, 2024. Keywords were chosen to limit the search outcomes to subjects of “circulating-free DNA” and “healthy”. The final search query was “cfDNA concentration” AND “healthy controls” AND “ng/mL” AND “plasma”. The returned studies were first assessed by title and abstract. Reviews and systematic reviews, inaccessible articles, and conference/presentation abstracts were excluded first, as they did not have any primary data, as well as non-English articles.

Studies that moved to full-text review were evaluated by the following criteria in order: subjects were human and healthy, measurements were in ng/mL, plasma cfDNA was measured, and mean and standard deviation were reported. Studies in which cfDNA concentration was reported exclusively in non-mass units (e.g., genome equivalents per milliliter, copies per milliliter, or Cq values without a clearly described calibration curve allowing conversion to ng/mL) were excluded, because these outputs are platform and assay dependent and not directly comparable across quantification methods. This restriction to ng/mL was intended to minimize additional analytical heterogeneity introduced by differing PCR calibration schemes and unit definitions across instruments and laboratories. Articles that met all these criteria (*n* = 35) were also checked if they included measurements for each subject in the study for subsequent analysis. Two investigators (AD and IG) independently reviewed the results from the literature search to determine inclusion in this study. Reviewers disagreed with selection results for some studies. Disagreements were defined as any instance in which at least one reviewer said yes and another said no to inclusion, or one could not find the study. The two investigators went through the articles with disagreements together for a second time in order to reconcile them.

### 2.2. Data Extraction and Assessment

The following data for included studies were independently extracted by AD and IG into an electronic table: if the blood collection vial contained EDTA, DNA extraction kit and company, DNA quantification method and kit, reference gene (if PCR was used), number of healthy patients, mean age of subjects, and mean and standard deviation of cfDNA.

A formal risk of bias assessment tool, such as those designed for interventional or outcome-based clinical studies (e.g., Cochrane RoB 2.0 or ROBINS-I), was not applied in this review. This is because our study did not involve evaluation of interventions or clinical outcomes. Instead, we focused on the methodological characteristics of cfDNA quantification in healthy individuals. Thus, a quality assessment was performed on the included articles based on the methodological factors of interest: the number of patients in the healthy cohort of the study (at least 10 and at least 30 subjects), blood collection method (EDTA-coated vial or not), the DNA isolation method (whether a Qiagen kit was used), the DNA quantification method (if either PCR or Qubit was used or not), and if the article contained subject-level data. AD and IG independently performed the quality assessment by marking “yes” or “no” to the corresponding items in each category of a quality assessment table. Instances of disagreement were discussed, and a final rating was given.

No formal certainty or confidence assessment (such as GRADE) was conducted because this review did not evaluate clinical outcomes or affect sizes of interventions. The aim of the review was to characterize methodological variability in cfDNA measurement among healthy individuals rather than to synthesize evidence for a specific clinical effect. As such, assessing certainty in a traditional evidence-to-decision framework was not applicable.

A GitHub repository was made containing the details of all article data collected and pre-processed for this review. The repository can be accessed via https://github.com/aaronn107/cfDNA-Systematic-Review (accessed on 18 December 2025).

### 2.3. Methodology Flow Diagram

A flow diagram was created to aggregate the mean, standard deviation, and number of studies in different methodological pathways. The data were first split into PCR (qPCR, rtPCR, etc.) and fluorometric quantification (specifically Qubit), as specified in each article’s methods. We excluded the few studies’ fluorometric quantification where the specific method was not specified from this analysis. These were then each split to show studies that also used Qiagen or other kits, denoted as “other”, referring to kits used that are not Qiagen. The equation used for combining the means of studies is

(1) $$M_{k}=\frac{\sum_{i=1}^{k}x_{i}*n_{i}}{\sum_{i=1}^{k}n_{i}}$$

where *k* is the number of groups, *x_i_* is the mean of the *i*th group, and *n_i_* the number of studies in the *i*th group [16]. The equation used for the pooled standard deviation is

(2) $$S_{k}=\sqrt{\frac{\sum_{i=1}^{k}s_{i}^{2}*{(n}_{i}-1)}{(\sum_{i=1}^{k}n_{i})-k}}$$

where *k* is the number of groups, *s_i_* is the standard deviation of the *i*th group, and *n_i_* the number of studies in the *i*th group [17].

### 2.4. Multiple Regression Analysis

To determine which factor in the healthy patient cfDNA analysis pipeline is most significant, a multiple linear regression was carried out by fitting a binary linear model to the data using R. This was carried out to discover statistically significant variables that impact the cfDNA values of the included studies. The variables included for this regression are whether or not the vials were EDTA coated, the extraction kit company used, and whether PCR was used. We then split the data into studies that used PCR and studies that did not use PCR. A multiple linear regression analysis was performed on the two datasets provided by this split. The goal was to discover if there were different significant variables impacting the cfDNA yields of the PCR studies as opposed to the non-PCR studies.

### 2.5. Box and Whisker Plots

Box plots were created in R to display the mean and standard deviations of different groups, enabling comparison of PCR vs. non-PCR studies. Box plots excluding custom kits were also created to review the impact that this has on the spread of data. Custom kits refer to included studies in which the authors used their own non-standard isolation kit. No kit refers to included studies in which the authors made no mention to the isolation method used.

## 3. Results

### 3.1. Literature Search Results

**Figure 1:**
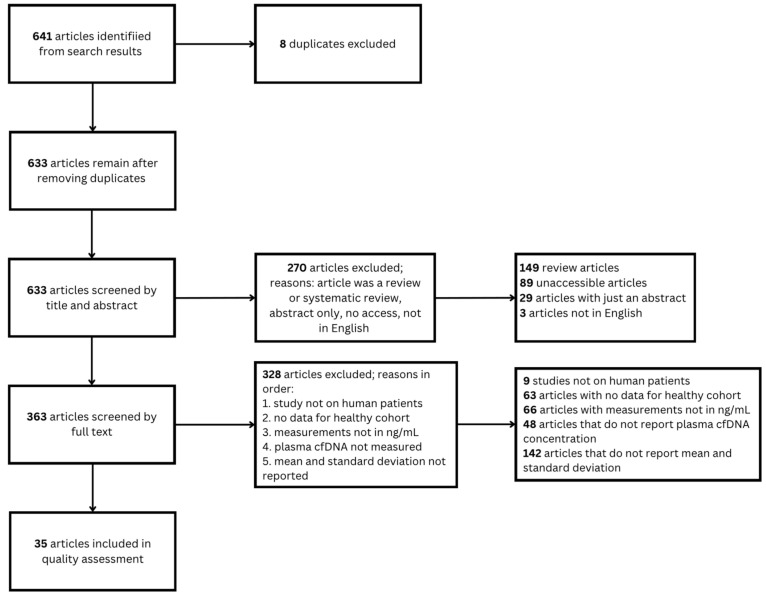
PRISMA flow diagram of the search strategy. A total of 35 studies were eligible for the review and quality assessment.

The literature search process is shown in Figure 1. A total of 641 articles were obtained via database search and 633 remained after removing duplicates. After screening these articles by title and abstract, 270 were removed for one of the following reasons: article was a review or a systematic review (*n* = 149); article was inaccessible (*n* = 89); abstract only (*n* = 29); or article was not in English (*n* = 3). Of the 363 articles that moved onto full-text review, 328 articles were removed for one of the following reasons: study was not on human subjects (*n* = 9); no data for a healthy cohort were reported (*n* = 63); measurements were not in ng/mL (*n* = 66); plasma cfDNA concentration was not reported (*n* = 48); or mean and standard deviation were not reported (*n* = 142). Thus, the results of 35 articles were used in subsequent quality assessment and analysis. Six of these articles included subject-level data, which were also used for analysis. The exclusion reasons for all other articles are included in Table S1–Master List available at https://github.com/aaronn107/cfDNA-Systematic-Review (accessed on 18 December 2025).

### 3.2. Quality of Included Articles

The results of the quality assessment are shown in Supplementary Table S1, it contains publications [14,18,19,20,21,22,23,24,25,26,27,28,29,30,31,32,33,34,35,36,37,38,39,40,41,42,43,44,45,46,47,48,49,50,51]. Only three articles fulfilled all of the quality criteria. No single criterion was met by all articles. Only six articles contained subject-level data. More than half of the studies used a Qiagen kit to isolate cfDNA from plasma (*n* = 22; ~63%), and almost all studies used vials containing EDTA for blood collection (*n* = 31; ~89%), as well as either PCR or Qubit to quantify cfDNA (*n* = 28; 80%). Additionally, 19/21 of the articles that used a Qiagen kit to isolate cfDNA used either PCR or Qubit to quantify. Multiple articles consisted of more than one experiment and reported more than one set of measurements [14,18,19,20,21,22,23,24]. However, only one experiment per study was included in the quality assessment table for simplicity. Detailed summary statistics and specific methodological characteristics for each included study are provided as Supplementary Material (Table S1–Included Articles) found in https://github.com/aaronn107/cfDNA-Systematic-Review (accessed on 18 December 2025).

### 3.3. Methodology Flow Diagram

**Figure 2:**
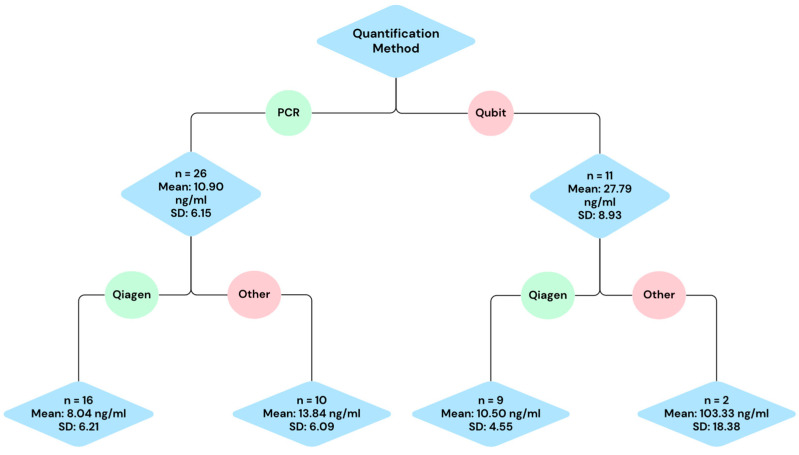
Flow diagram showcasing different methodology pathways seen in the selected articles. Each pathway showcases the aggregated mean, pooled standard deviation (SD), and the number of studies that used the pathway (*n*).

The flow diagram showcases the cfDNA means resulting from different methodology pathways (Figure 2). Some articles contained multiple datasets with different means. Thus, in this diagram, the number of studies refers to the number of datasets with a resulting mean. The combined mean of the 26 PCR-only datasets is 10.90 ± 6.15 ng/mL while the combined mean of the 11 Qubit datasets is 27.79 ± 8.93 ng/mL. The pathway of using PCR and Qiagen kits results in a combined mean of 8.04 ± 6.21 ng/mL with 16 studies included. The pathway of using PCR and other (non-Qiagen) kits results in a combined mean of 13.84 ± 6.09 ng/mL with 10 included datasets. The pathway of using Qubit and Qiagen kits results in a combined mean of 10.50 ± 4.55 ng/mL with nine included datasets. The pathway of using Qubit and other (non-Qubit) kits results in a combined mean of 103.33 ± 18.38 ng/mL with two included datasets. The PCR and Qiagen kit pathway gave the lowest combined mean and standard deviation. This pathway also had the highest sample size, with 16 studies included compared to 10, 9, and 2. Meanwhile, the Qubit and other kit pathway gives a combined mean and standard deviation that is much higher than the other pathways of methodology. Because of this, statistical methods were used to explore the reasoning behind this large range in cfDNA mean and standard deviation.

### 3.4. Statistical Analysis

The multiple linear regression analyses showed that the only statistically significant variables in the data are whether PCR was used (*p* = 0.01), and whether the kit used was custom made (*p* = 0.0498). All other variables were found to agree with the null hypothesis of not having an effect on the mean cfDNA. The data were then split into PCR and non-PCR subsets for further analysis and examination into this significant variable.

After splitting the data, another multiple linear analysis was performed. The variables included were whether the vial is EDTA coated, whether the kit was Qiagen, and whether the number of healthy patients included in the study was significant. Using EDTA-coated vials or a Qiagen kit showed no statistical significance in either the PCR dataset or the non-PCR dataset. The number of healthy patients used did have significance in the PCR data. Using 40 or more study participants compared to using less than 40 had a *p*-value of 0.02, showing that the number of healthy samples in these studies influenced the cfDNA mean values.

**Figure 3:**
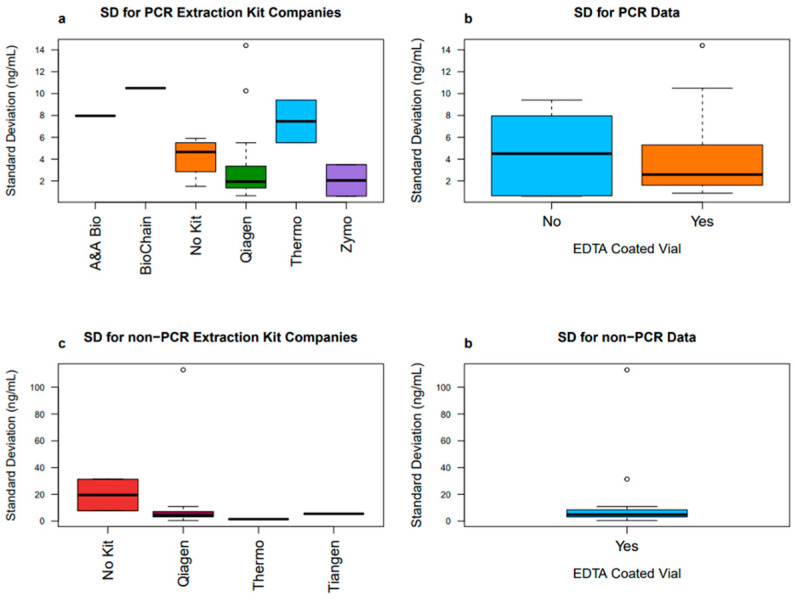
Box plots comparing the standard deviation of cfDNA values for included articles. Custom kits are excluded from the calculations and both PCR and non-PCR box plots are compared. The circled points represent outliers within the data.

**Figure 4:**
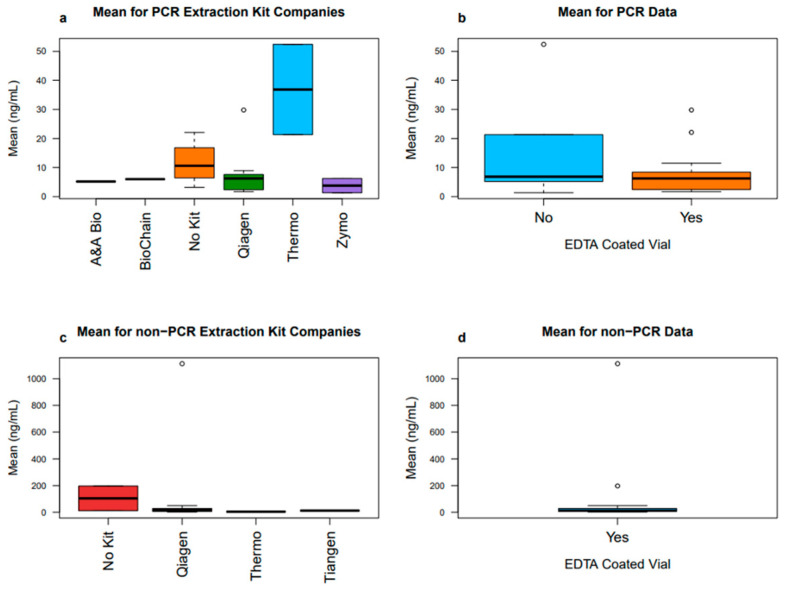
Box plots comparing the means of cfDNA values for included articles. Custom kits are excluded from the calculation and both PCR and non-PCR box plots are compared. The circled points represent outliers within the data.

To further examine the effects of different cfDNA processing steps on the variability of results, we plot the respective distributions of mean and standard deviation values of the data. These boxplots were split into PCR and non-PCR subsets and further faceted by the DNA isolation method or by the blood collection vial type. There were also few included studies that used custom extraction kits and did not use PCR. More boxplots were created excluding the custom kits to see its impact on cfDNA mean and standard deviation values (Figure 3 and Figure 4).

After excluding the custom kits, it can be seen that the remaining studies all use EDTA-coated vials. It is important to note that none of the PCR studies used custom extraction kits. For the non-PCR studies, not using an extraction kit had the highest variability in both cfDNA means and standard deviations. However, the spread in standard deviation for all extraction kits is dramatically lower than the previous box plot showing that removing custom kits eliminates this high variability (Figure 3c). Removing custom kits had no impact on the EDTA vial coating status when looking at standard deviation, indicating that the custom extraction kits contributed to the large variability in the previous plots (Figure 3d). After excluding custom kits, the mean values in the non-PCR studies have a very low variability in Qiagen, Thermo Fisher, and Tiangen kits, with the largest variability now being seen in the studies that used no kits (Figure 4c). The custom kits, or no kits, thus have high variation in the resulting cfDNA mean and standard deviation values. Commercial extraction kits yield a much lower variation and showcase more consistency in both the means and standard deviations. This is not surprising, given the lack of standardization that occurs when commercial kits are not used.

There were five articles that included individual data with their paper. These data were analyzed (see Supplementary S1) and follow the trends and statistics of the aggregated data.

## 4. Discussion

The goal of this review was to summarize reported ranges of circulating cell-free DNA (cfDNA) levels in healthy individuals across commonly used methodological pipelines, and to make recommendations of methodologies that result in less variability, to inform and aid future early-detection algorithms. The ranges of expected cfDNA values given certain methodological pathways are provided in Figure 2. Among studies using PCR quantification with a Qiagen kit, the aggregated mean cfDNA concentration was 8.04 ± 6.21 ng/mL. The expected cfDNA value when using PCR quantification and non-Qiagen kits is 13.84 ± 6.09 ng/mL. The expected cfDNA value when using Qubit quantification coupled with a Qiagen kit is 10.50 ± 4.55 ng/mL. Lastly, the expected cfDNA range when using Qubit quantification and a non-Qiagen kit is 103.33 ± 18.38 ng/mL. A multiple linear regression analysis identified PCR usage as the most significant factor affecting mean cfDNA values (*p* = 0.01), suggesting that amplification-based methods introduce variability in cfDNA quantification. Given this, we separated PCR and non-PCR datasets for further evaluation.

Among PCR-based studies, including ≥40 healthy subjects significantly reduced cfDNA variability (*p* = 0.02), indicating that larger sample sizes yield more stable cfDNA means, most likely as a result of a larger sample size. Custom extraction kits introduced greater variability in cfDNA measurements (*p* = 0.0498), which suggests that non-standardized methods may be less reliable.

Our findings align with previous reviews, which have reported substantial variability in cfDNA concentrations due to differences in pre-analytical handling and extraction techniques. The only pre-analytical variable we assessed was whether the blood collection tube was EDTA coated, which was found to be insignificant in relation to the mean and standard deviations (*p* > 0.05). For non-PCR studies, the only studies with non-EDTA-coated tubes also used custom kits. These studies had much higher mean and standard deviations than non-PCR studies that used EDTA-coated tubes and non-custom kits (fluorometric). In contrast, PCR studies had ranges of mean and standard deviation values that were not so different from each other, although the spread of non-EDTA-coated tube studies was larger than that of EDTA-coated tube studies. This shows that customs kits have a great effect on increasing the mean cfDNA measured, aligning with the multiple linear regression analysis that showed that custom kits had a significant effect on measured cfDNA (*p* = 0.0498).

We found that the type of cfDNA isolation kit did not significantly impact mean cfDNA concentrations, despite widely held assumptions about variability across commercial kits. This is particularly noteworthy given that Qiagen kits were the most frequently used in our dataset (*n* = 22; ~63%) and have been widely recommended in the previous literature for their performance and reproducibility. For example, Mauger et al. [13] and Polatoglou et al. [52] both reported that the QIAamp Circulating Nucleic Acid Kit (Qiagen) yielded high cfDNA recovery with low variability when compared to other commercial and custom kits. Similarly, Markus et al. [53] also found that the QIAamp Circulating Nucleic Acid Kit (Qiagen) produced the highest median yield and low variability among seven commercial kits.

However, our analysis did not identify a statistically significant difference in cfDNA yield between Qiagen and non-Qiagen kits. This may be due to high inter-study heterogeneity, small subgroup sample sizes, or confounding from other methodological differences such as quantification techniques or other pre-analytical variables that we did not account for. Moreover, a systematic review by Trigg et al. [10] emphasized that as long as manufacturers’ instructions are followed, the specific choice of commercial extraction kit may not critically impact results, aligning with our finding that kit brand alone may not explain observed variation.

It is important to emphasize that the dataset included a limited range of commercial extraction kits, largely reflecting the period and design of the underlying studies, many of which pre-date the most recent wave of cfDNA kit development. More recent products from multiple manufacturers (including newer Qiagen kits and competitor platforms) offer lower cost, higher throughput, and automation-ready workflows, and many are benchmarked internally against the QIAamp Circulating Nucleic Acid Kit as a legacy reference. Our data therefore support the use of a well-validated commercial kit to reduce variability, but they do not allow us to conclude that any single vendor or product is superior to all current alternatives. In practice, laboratories should select an extraction kit that is validated in their own setting (including automated formats where relevant) and report sufficient methodological detail to enable comparison across platforms. 

In contrast to the extraction kit, the quantification method—specifically the use of qPCR—was a significant factor in cfDNA mean concentration in our review (*p* = 0.01). This aligns with prior work by Mauger et al. [13], who compared fluorometric (Qubit) and qPCR-based quantification methods. Their study found that qPCR had improved sensitivity at low cfDNA concentrations, particularly using assays that target highly repetitive genomic elements like Kpn. However, they also reported that Qubit was sufficient and even preferable in scenarios where DNA input was low, especially due to its cost efficiency and practicality. A similar finding was reported by Till et al. [54] which found that there was no significant difference in coefficients of variation, as well as high correlation between the use of qPCR, Qubit, or ddPCR to measure cfDNA in healthy patients.

Likewise, Trigg et al. [10] concluded that both qPCR and fluorometric methods yielded comparable cfDNA measurements but emphasized that fluorometry may be more suitable for cost-effective screening, while qPCR offers higher sensitivity and specificity, especially for downstream applications like fragment analysis. Given that qPCR use was significantly associated with cfDNA mean values in our dataset, qPCR appears to be a sensitive and influential quantification approach in healthy-control studies.

Taken together, these findings underscore the complexity of cfDNA measurement and highlight that while some methodological variables (e.g., kit brand) may not independently affect cfDNA means, others (e.g., quantification approach) can exert significant influence. The literature most frequently reports lower variability with the use of EDTA-coated vials (as recommended by Trigg et al. [10]), the QIAamp Circulating Nucleic Acid Kit for cfDNA isolation (given its widespread use and demonstrated reproducibility), and qPCR-based quantification for its sensitivity and significant influence on cfDNA levels

The main limitation of this review is the small number of included studies, coupled with heterogeneity in study designs. This stems from the under-reporting of healthy control information. Of 640 articles, only 35 articles met all inclusion criteria and were included in the analysis. Heterogeneity in study design meant that there was a small sample size per subgroup, particularly regarding extraction kit companies, custom kits, and PCR primers used. This reduced statistical power, possibly leading to certain factors appearing more or less significant than they truly are. For example, our study showed that PCR quantification had a significant effect on mean cfDNA measured, despite previous studies showing that PCR and Qubit measurements are correlated and that Qubit is also a recommended quantification method that is highly sensitive and accurate [10,11].

Due to the small sizes, the significance of different PCR primers/reference genes was not assessed, even though it would have been interesting to consider these effects (β-actin, long interspersed element-1 (LINE-1), ALU, etc.). Shen et al. [14] notes that while some reference genes like β-actin and LINE-1 can be expressed stably, their copy numbers per genome differ significantly, increasing variability in measurements. ALU tandem repeats or other non-coding segments can be a more favorable reference gene because they are a more abundant and stable target. Thus, while our study found that PCR significantly affects mean cfDNA in healthy patients, future research should further investigate the optimal reference gene that ensures both accuracy and minimal variability in cfDNA quantification.

A limitation of our review is the absence of data on automated magnetic extraction instruments (e.g., KingFisher Flex, AutoPure 24, QIAsymphony). None of our included studies explicitly reported using automated magnetic separation-based systems. This likely reflects that healthy control cohorts are often characterized in research settings where manual extraction predominates. However, automated extraction is increasingly adopted in high-throughput clinical laboratories and offers potential advantages including reduced hands-on time, improved reproducibility, and greater accuracy as noted by Polatoglou et al. [52].

## 5. Conclusions

This systematic review underscores the impact of methodological variability on cfDNA quantification in healthy controls and highlights key factors influencing yield, particularly PCR use, sample size, pre-analytical handling, and custom extraction kits. While commercial kits offer consistency, significant variability remains, emphasizing the need for standardized cfDNA protocols in research and clinical applications. Based on this analysis and previous studies, we recommend using EDTA-coated vials, a standardized commercial extraction kit, and qPCR for quantification, as these methods were associated with lower variability and more consistent cfDNA recovery in our dataset. Additionally, we provide a reference of expected means and standard deviations of cfDNA in healthy controls for methodological pathways by aggregating such measurements from the included studies (Figure 2). While it can be made more robust through the inclusion of a higher sample size of measurements in the future, these aggregated values provide a descriptive reference for cfDNA concentrations reported in healthy controls under commonly used methodological conditions. Overall, our findings should be interpreted as descriptive and hypothesis generating rather than definitive standards, given the heterogeneity and limited number of eligible studies.

## Data Availability

All data generated or analyzed during this study are included in this published article and its Supplementary Information files. The detailed Supplementary Material for this article, including the details of all data collected and processed for this review, can be found at https://github.com/aaronn107/cfDNA-Systematic-Review (accessed on 18 December 2025).

## Supplementary Materials

The following supporting information can be downloaded at https://www.mdpi.com/article/10.3390/diagnostics16060821/s1, Figure S1: Box plots comparing the standard deviation of cfDNA values for included articles when using PCR only or non-PCR methods. Figure S2: Box plots comparing the mean of cfDNA values for included articles that used PCR only and non-PCR methods. Table S1: Quality assessment table. Supplementary S1: Individual Level Data.

## Abbreviations

The following abbreviations are used in this manuscript:

cfDNA	Circulating cell-free DNA
PRISMA	Preferred Reporting Items for Systematic Reviews and Meta-Analyses
qPCR	quantitative PCR
dPCR	digital PCR
