# Supplementary material for: Systematic Review of Methods for Measuring Circulating Cell-Free DNA in Plasma of Healthy Individuals
Source: Diagnostics (Basel). 2026 Mar 10;16(6):821. doi: 10.3390/diagnostics16060821 (PMC13025540; doi:10.3390/diagnostics16060821)
Supplement: Supplementary file 1 [file diagnostics-16-00821-s001.zip › diagnostics-4081669-supplementary.pdf]

#	Citation	>= 10 subjects	>= 30 subjects	EDTA Vial	Qiagen Kit	PCR or Qubit	Individual Data
1	Alghofaili et al., 2019 [25]	Yes	Yes	Yes	Yes	Yes	Yes
2	Breitbach et al., 2014 [18]	Yes	Yes	Yes	No	Yes	No
3	Chen et al., 2021 [26]	Yes	Yes	Yes	Yes	Yes	Yes
4	Chu et al., 2024 [27]	Yes	Yes	Yes	Yes	No	No
5	Cifuentes et al., 2023 [28]	No	No	Yes	No	No	Yes
6	Dopico et al., 2022 [19]	No	No	Yes	Yes	Yes	No
7	Ershova et al., 2017 [29]	Yes	Yes	No	No	No	No
8	He et al., 2016 [20]	Yes	No	Yes	Yes	Yes	No
9	Hussein et al., 2019 [21]	Yes	No	Yes	Yes	Yes	No
10	Jiang et al., 2018 [30]	Yes	Yes	Yes	No	Yes	Yes
11	Le Calvez-Kelm et al., 2016 [31]	Yes	No	Yes	Yes	Yes	No
12	Lin et al., 2018 [32]	Yes	Yes	Yes	Yes	No	No
13	Maggi et al., 2018 [33]	No	No	Yes	Yes	Yes	No
14	Malinovskaya et al., 2019 [34]	Yes	No	No	No	No	No
15	Marchio et al., 2018 [35]	Yes	Yes	Yes	No	Yes	No
16	Mattox et al., 2023 [36]	Yes	Yes	Yes	No	Yes	Yes
17	Mazurek et al., 2016 [37]	Yes	No	No	No	Yes	No
18	Miao et al., 2019 [38]	Yes	Yes	Yes	No	Yes	No
19	Myint et al., 2018 [39]	Yes	Yes	Yes	Yes	Yes	No
20	Palande et al., 2022 [40]	Yes	No	Yes	Yes	Yes	No
21	Kim et al., 2012 [41]	Yes	Yes	Yes	Yes	Yes	No
22	Qi et al., 2024 [22]	Yes	Yes	Yes	Yes	Yes	Yes
23	Salimi and Burkhani, 2019 [42]	Yes	Yes	Yes	Yes	Yes	No
24	Shen et al., 2022 [14]	Yes	Yes	No	No	Yes	No
25	Sinha et al., 2019 [23]	Yes	Yes	No	No	Yes	No
26	Szpechcinski et al., 2009 [43]	Yes	No	Yes	Yes	Yes	No
27	Szpechcinski et al., 2015 [44]	Yes	Yes	Yes	Yes	Yes	No
28	Szpechcinski et al., 2016 [45]	Yes	No	Yes	Yes	Yes	No
29	Tug et al., 2014 [46]	Yes	Yes	Yes	No	Yes	No
30	Ackerveken et al., 2023 [47]	No	No	Yes	Yes	Yes	No
31	Wu et al., 2019 [48]	Yes	Yes	Yes	Yes	Yes	No
32	Wu et al., 2022 [49]	Yes	Yes	Yes	Yes	Yes	No
33	Yuwono et al., 2021 [24]	Yes	Yes	Yes	Yes	Yes	No
34	Zhang et al., 2014 [50]	Yes	Yes	Yes	No	No	No
35	Zhong et al., 2020 [51]	Yes	Yes	Yes	No	No	No

Table S1: Quality Assessment Table

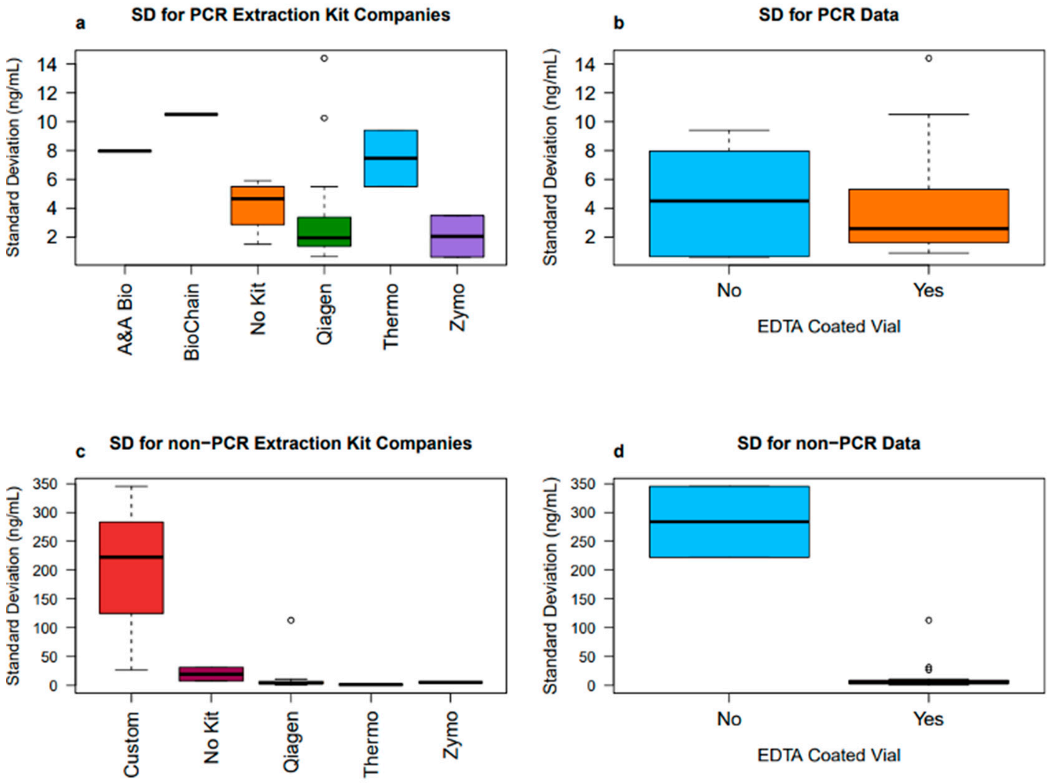

Figure S1: Box plots comparing the standard deviation of cfDNA values for included articles when using PCR only or non-PCR methods. Custom kits are included in this analysis.

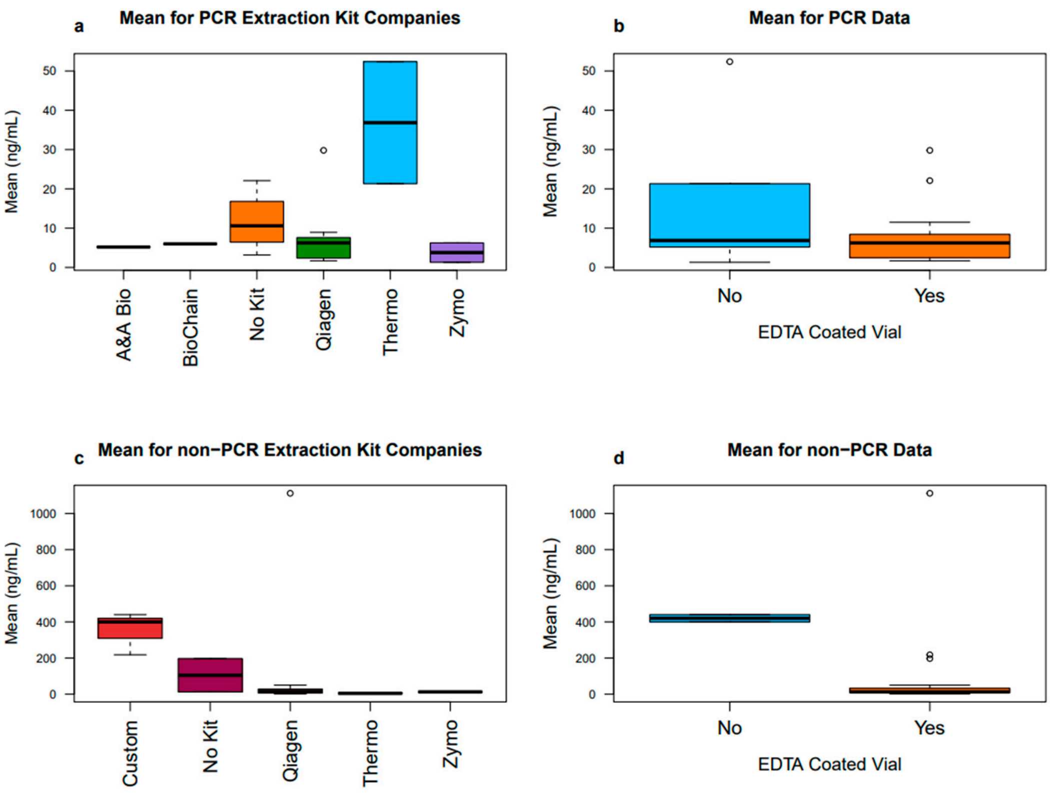

Figure S2: Box plots comparing the mean of cfDNA values for included articles that used PCR only and non-PCR methods. Custom kits are included in this analysis.

For both the mean and standard deviation for the PCR data, Thermo Fisher kits had the largest variation in both the mean and standard deviation when comparing extraction kits (Figure S1a and Figure S2a). Qiagen and Zymo-Research kits had the lowest variability in both the mean and standard deviation (Figure S1a and Figure S2a). Not using an EDTA coated vial resulted in a larger spread compared to using it (Figure S1b and Figure S2b). The standard deviation for the non-PCR studies that did not EDTA coat their vials also had a much larger spread than the EDTA coated (Figure S1d). Meanwhile the mean values of the non-PCR studies had similar spreads regardless of EDTA vial coating (Figure S1d). The few included studies that used custom extraction kits also did not use PCR. More boxplots were created excluding the custom kits to see its impact on cfDNA mean and standard deviation values (Figure 3 and Figure 4).

Supplementary S1
Individual Level Data

There were 5 articles that included individual data with their paper. One of these articles contained two data sets and had two groups of healthy people. Three of the studies used Qubit DNA quantifications

methods along with Qiagen kits to quantify their cfDNA samples. The mean of their cfDNA samples is 9.84 $\pm$ 5.19 ng/ml. The aggregated mean of cfDNA of all of the studies in this review using this methodology pathway is 10.50 $\pm$ 4.55 ng/ml. The mean found from the individual data is similar to the aggregated mean found in the flowchart created (Figure 2). One of the articles containing individual data used PCR DNA quantification followed by a non-Qiagen kit. The mean of these cfDNA values is 5.97 $\pm$ 10.45 ng/ml. This is roughly similar to the aggregated mean of all included studies that used this methodology pathway, 13.84 $\pm$ 6.09 ng/ml (Figure 2).
